# Complexity vs linearity: relations between functional traits in a heterotrophic protist

**DOI:** 10.1186/s12862-022-02102-w

**Published:** 2023-01-11

**Authors:** Nils A. Svendsen, Viktoriia Radchuk, Thibaut Morel-Journel, Virginie Thuillier, Nicolas Schtickzelle

**Affiliations:** 1 Earth and Life Institute, Biodiversity Research Center, Université Catholique de Louvain, Louvain-La-Neuve, Belgium; 2 Leibniz Institute for Zoo and Wildlife Research (IZW), Alfred-Kowalke-Straße 17, 10315 Berlin, Germany; 3 Centre Interdisciplinaire de Recherche en Biologie (CIRB), Collège de France, PSL Research University, CNRS UMR 7241, Paris, France

**Keywords:** Functional traits, Linearity assumption, Soft/hard traits framework, *Tetrahymena thermophila*, Trait relations

## Abstract

**Background:**

Functional traits are phenotypic traits that affect an organism’s performance and shape ecosystem-level processes. The main challenge when using functional traits to quantify biodiversity is to choose which ones to measure since effort and money are limited. As one way of dealing with this, Hodgson et al. (Oikos 85:282, 1999) introduced the idea of two types of traits, with soft traits that are easy and quick to quantify, and hard traits that are directly linked to ecosystem functioning but difficult to measure. If a link exists between the two types of traits, then one could use soft traits as a proxy for hard traits for a quick but meaningful assessment of biodiversity. However, this framework is based on two assumptions: (1) hard and soft traits must be tightly connected to allow reliable prediction of one using the other; (2) the relationship between traits must be monotonic and linear to be detected by the most common statistical techniques (e.g. linear model, PCA).

**Results:**

Here we addressed those two assumptions by focusing on six functional traits of the protist species *Tetrahymena* *thermophila*, which vary both in their measurement difficulty and functional meaningfulness. They were classified as: easy traits (morphological traits), intermediate traits (movement traits) and hard traits (oxygen consumption and population growth rate). We detected a high number (> 60%) of non-linear relations between the traits, which can explain the low number of significant relations found using linear models and PCA analysis. Overall, these analyses did not detect any relationship strong enough to predict one trait using another, but that does not imply there are none.

**Conclusions:**

Our results highlighted the need to critically assess the relations among the functional traits used as proxies and those functional traits which they aim to reflect. A thorough assessment of whether such relations exist across species and communities is a necessary next step to evaluate whether it is possible to take a shortcut in quantifying functional diversity by collecting the data on easily measurable traits.

**Supplementary Information:**

The online version contains supplementary material available at 10.1186/s12862-022-02102-w.

## Background

Biodiversity is declining at an alarming rate [1–5], requiring more than ever to be carefully measured in different ecosystems. Traditionally the focus when measuring biodiversity was on taxonomical diversity, e.g. species richness or evenness. However, such an approach has been criticized for its inability to bring a mechanistic understanding of the effects that species composing the community have on ecosystem functioning [1, 6, 7]. As an alternative, measuring functional diversity was suggested, whereby one measures the functional traits of organisms, defined as characteristics of an organism’s phenotype that affect its performance at the individual level [8], on the one hand, and that contribute to ecosystem-level processes, on the other hand [9–12]. Originally studies measuring functional diversity focused on mean values of functional traits per species. However, with the increasing recognition of the importance of intraspecific trait variation [13–15], functional traits are, over the last decades, increasingly measured at the level of the organism, i.e. they are measured on a sample of individuals instead of using species averages [16]. However, regardless of whether one measures functional diversity at the community or at a single species level, the challenges remain analogous.

**Fig. 1:**
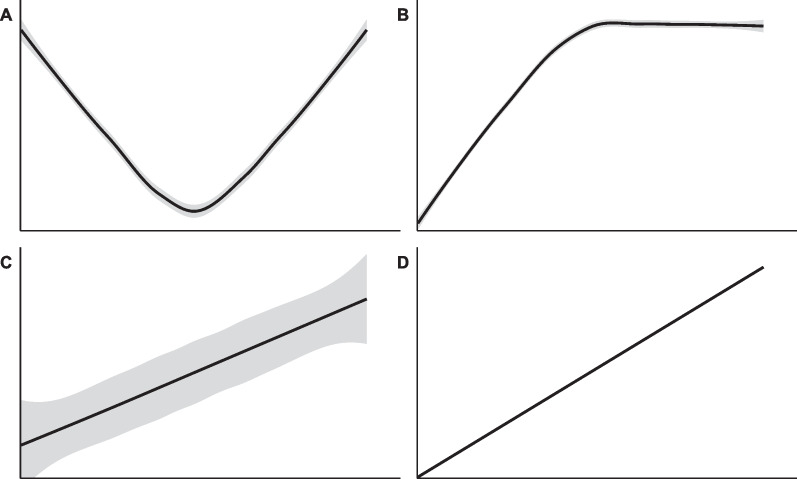
Four scenarios illustrating a spectrum of possible relations between hard and soft traits: **A** This non-monotonic relationship allows prediction of the hard trait using the soft one, but not through a simple linear method. **B** An example of a monotonic relation, where the two traits are linearly related on a portion of their variation domain, only allowing accurate predictions of one trait by the other (either way) on this part. **C** The traits are here linearly related, but reliable predictions cannot be achieved because of the high standard error. **D** The ideal linear, strong and monotonic relationship needed for PCA and correlations. Thus, one can use a trait as a proxy for another one only if there is a well-known relationship that is correctly estimated, implying (1) the knowledge of the form of the relationship between the two traits, (2) a relationship where the values of one trait change with the values of the other (i.e. no constant values of one trait as the other one is changing) because such a relation prevents prediction on one of the traits and (3) a standard error on the model parameter small enough to give a reliable prediction

The main challenge when measuring functional diversity within a species relates to the choice of which functional traits to measure. There are too many traits to measure them all, and efforts are limited, thus usually only a subset of possible traits are chosen [17, 18], often those that are rather easier to measure, like morphological traits [19–21]. However, the measured traits must be good proxies for both the organism’s fitness and its effects on ecosystem functioning. As one way of dealing with this, Hodgson et al. (1999) introduced the idea of soft and hard traits, where the former ones are relatively easy and quick to quantify, while the latter ones are more functionally meaningful but harder to measure. Ideally, we would measure hard traits (e.g. metabolic or physiological traits) when quantifying functional diversity, but since they are by definition difficult to measure, one could instead measure soft traits (e.g. leaf area) that are assumed to be linked to these hard traits. Such use of the soft traits as proxies for hard ones is promising but it is based on a strong assumption: hard and soft traits must be tightly connected. The situation for many species is likely that reasonable knowledge exists in the literature to make an informed selection of soft traits that could be proxies for hard traits, but exact relations between them are not yet established. Another implicit assumption lurking behind the most common statistical methods used to look for relations among variables (e.g. Pearson correlation, PCA) is that the relation between them is monotonic and linear (Fig. 1). However, these assumptions that a relation exists and is linear are rarely checked, and proxies are likely often taken for granted.

Here we tested the use of the soft/hard framework, and its underlying assumptions, by focusing on *Tetrahymena thermophila*, a unicellular ciliate that has widely been studied as a model system in cellular and molecular biology for more than 80 years and in ecology and evolutionary biology for over a decade [22–24]. Over these years, numerous studies provided a lot of information about *T. thermophila* metabolism [23, 25–28], reproduction [29–31], movement [32, 33] and morphology [24], allowing us to formulate the predictions about the expected relations between the soft and hard traits. Besides, this study system also allows us to carefully assess the existence of the expected relations as well as their linearity. Based on the knowledge synthesized from the literature and our own existing data, we have selected the following six traits of *T.* *thermophila* cells as functional: two morphological traits (cell size and shape), two movement traits (movement speed and trajectory tortuosity), oxygen consumption and population growth rate. In our experimental microcosm system, these traits vary in their functional meaningfulness and measurement difficulty: easy (morphological traits), intermediate (movement traits) and hard (oxygen consumption and population growth rate).

The morphological traits are considered functional because they relate to the resource use of *T. thermophilla*. Indeed, an increase in cell size is often a consequence of resource accumulation [34]. These resources could then be mobilized if environmental conditions become harsh. For example, when oxygen is present, the cells will produce and accumulate glycogen using a part of this oxygen [27, 28, 35]; glycogen is here used as a storage for energy and can be used to produce ATP (i.e. energy needed for the cell survival) through fermentation when oxygen is lacking [25], allowing cells to survive for few hours without respiring. The shape of the cell is an indicator of wellness for *T. thermophila* [34]. When the environment is stressful, *T. thermophila* cells tend to adopt a rounder shape, possibly because they exhaust all their metabolites (e.g. glycogen) in reserves to survive until the environmental conditions become suitable again. We classify these two morphological traits as “easy” since they remained indirect proxies of glycogen accumulation or wellness, and their quantification in our system only requires a snapshot picture of cells.

The two movement traits play a major role in resource foraging, hence survival, reproduction, and dispersal strategy, as shown in the very close species *T. pyriformis* [36, 37]. Swimming fast gives the advantage of quickly exploring space, allowing the cells to potentially find a better environment, at the cost of the energy needed to move and the risk of exhausting themselves to death. The same reasoning applies to the trajectory linearity, since a tortuous trajectory could enhance local foraging by maximizing resources exploited in the neighborhood, while a straighter trajectory will allow access to distant patches with possible better resources and escaping harsh local conditions. We considered these two movement traits as having an “intermediate” level both regarding the measurement difficulty, since measuring them requires recording a video with trajectories of moving cells, but also functionally, since they directly impact the foraging abilities of *T. thermophila* cells*.*

Regarding the last group of traits, oxygen consumption is a direct proxy of the cell metabolic rate, and one of the major factors driving heterotrophic protist community structure [38]. Population growth rate is directly proportional to the individual clonal cell reproduction rate and is the main driver of biomass production, which is often used as a proxy for ecosystem functioning or species wellness [39–44]. These two traits are the most difficult to measure in our microcosm system because they cannot be measured from a snapshot data recording (picture or video), which is possible to acquire even in the field, but instead involve a time series of measurements using specialized equipment in the lab. However, they are also more directly connected to the ecological parameters of the population (i.e. metabolism and biomass production), making their estimation very desirable in functional diversity studies. Thus, according to the soft/hard framework [45], if we detect a significant relationship between these hard traits and the intermediate/easy ones, it would allow for indirect estimation of these hard traits based on snapshot picture or video measurements, which are even possible in the field.

**Fig. 2:**
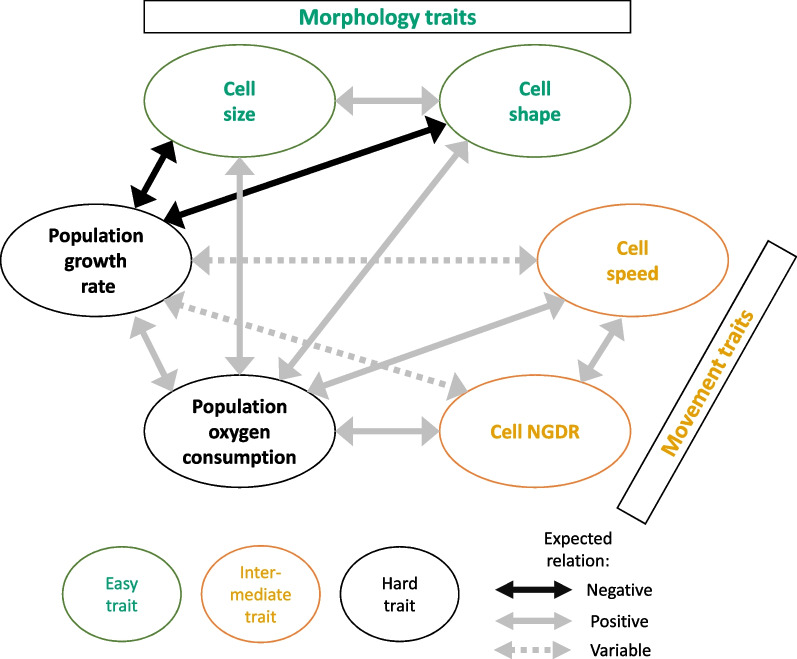
Overview of the relations between the selected functional traits of *T. thermophila* cells. Arrows indicate how each trait affects the other ones, with a black arrow indicating an expected positive relation, a grey arrow for a negative one, and a dotted grey arrow for relations that can vary based on the environment. Each trait is colored based on its measurement difficulty with (1) green for easy, (2) yellow for intermediate, and (3) black for hard

Based on the accumulated knowledge on the ecology of *T. thermophila*, we expect some of our chosen easy or intermediate traits to be linked with the hard traits. We expect that cell shape and cell size would have a negative relation with population growth rate, as the faster a strain reproduces, the less time its cells have to accumulate resources, to become longer and larger. The oxygen consumption rate is expected to have a positive relation with both movement traits and population growth rate since these processes require energy. However, the relation between movement traits and population growth rate might be variable, since movement is an energy-consuming process that is rewarded with obtained nutrients when the organism is foraging. We expect this relation to be positive in food-rich environments, and negative otherwise. Further, since bigger and waterdrop-shaped cells may be a sign of a higher metabolism, we also expect cell size and shape to be positively related to oxygen consumption. A complex set of relations can therefore be predicted (Fig. 2), including sometimes contradictory predictions (e.g. cell size and shape are expected to be negatively related to growth rate, but positively to oxygen consumption, itself expected to positively relate with growth rate). This is an illustration that despite considerable knowledge, predicting relations among traits is not an obvious task, reinforcing the interest in testing how assumed relations are indeed real.

We measured the above-described six functional traits on 40 genetically distinct *T. thermophila* strains (i.e. clonally reproducing genotypes), that differ in geographic origin and time since extraction from the field [46]. These strains were previously shown to exhibit clear differences in several life-history characteristics such as growth rate, maximum density, and survival under starvation conditions [46–48]; which have been demonstrated to be reliable phenotypic traits at the strain level because of the high repeatability of their measures through time [47–50]. Such use of several strains covers part of the existing intraspecific variation in *T. thermophila*, allowing testing for relations among the six functional traits. To explore the relations among the traits, we first looked for general trends between all the traits through a Principal Component Analysis (PCA). Secondly, we used General Additive Models (GAM) to test if the predictions were improved by considering the possibility that relations between the traits are non-linear. Specifically, we assessed the form and standard deviation (see Fig. 1) of the best fit, for all possible pairwise relations between the six traits, regardless of the difficulty of taking measurements.

## Results

**Fig. 3:**
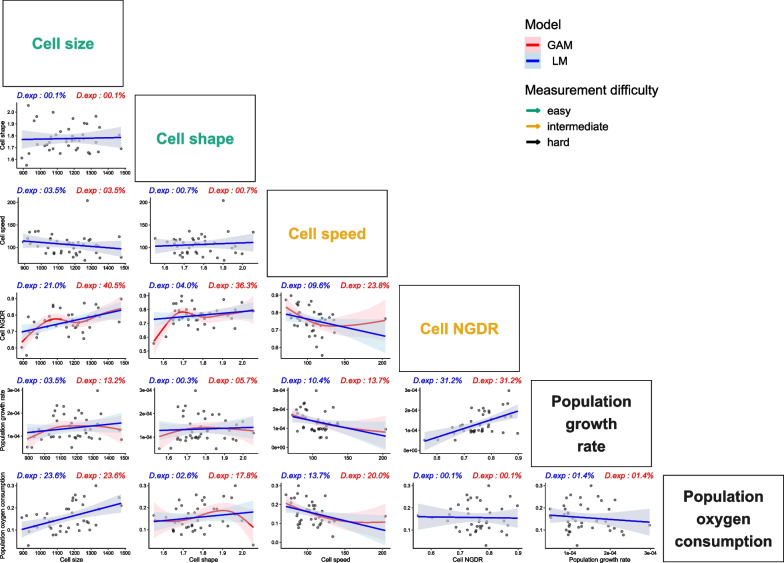
Pairwise relations among the six functional traits measured for the 40 T*. thermophila* strains. Each dot represents the average value of all replicates at the strain level, the blue line a linear model, and the red curve a GAM. Both are represented with their respective 95% confidence interval. Above every graph is displayed the deviance explained (D.exp) by each model. When displayed, the non-linear GAM is significantly better than the linear model (e.d.f. > 1). Otherwise, the non-linearity did not improve the model fit, both models remaining linear and identical (hence the same D.exp), and on the graph only the linear model is displayed

Among the 15 pairwise relations between the six functional traits, 8 were non-linear as evidenced by the higher deviance explained when the relation was permitted to be non-linear through GAMs (Fig. 3). Thus, considering the non-linearity significantly improved the model fit in half of the cases. However, for the other 7 relations, the simple linear model remained the best fit. Now, looking at the best model (linear or not) for the 15 relations, only 3 showed a deviance explained above 25%: both cell size (40.5%) and cell shape (36.3%) when predicting NGDR with a non-linear model, and NGDR itself predicting population growth rate (31.2%) with a linear model (Fig. 3). As the D14 strain seemed to have an important leverage on some relations (i.e. is a potential outlier), especially the ones involving cell speed, we also performed the analysis with that strain removed and still found 8 significant non-linear relations, and similar deviance explained across all 15 best-fitted models (See Additional file 1: Fig. S1). Overall, all models explained a limited proportion of the deviance, making the predictions based on a single trait quite unreliable (Additional file 2, Additional file 3). We also fitted linear models to log-transformed data, since data transformations are typically used to deal with non-linear relations, but that did not result in a better model fit compared to GAMs (See Additional file 5: Fig. S3).

**Fig. 4:**
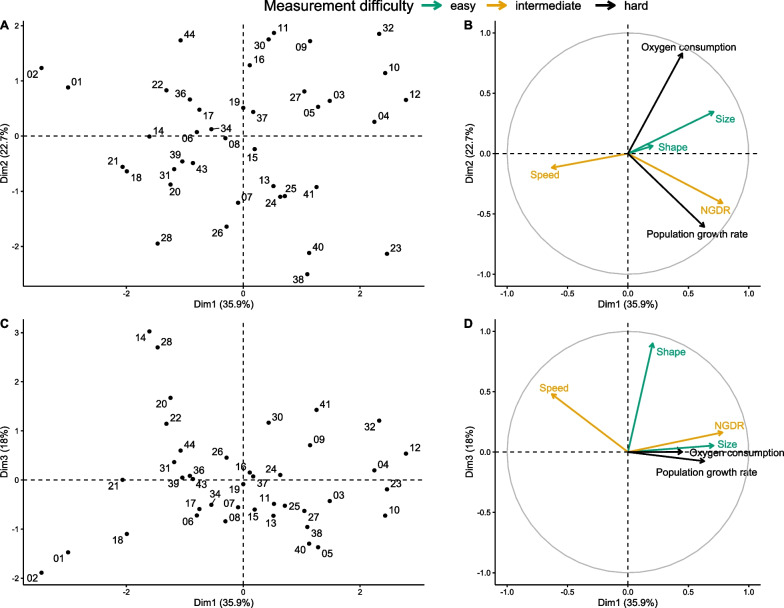
PCA analysis performed on the six functional traits (averaged at the strain level) for the 40 T*. thermophila* strains*.* The left panels represent the distribution of the strains along dimensions 1 and 2 (**A**) and along dimensions 1 and 3 (**C**). The numbers in (**A**) and (**C**) stand for the labels of the strains. The right panels represent the associated correlation circles along the first and second dimensions (**B**) and the first and third dimensions (**D**). The arrows assess the representativity of the traits on the considered dimensions, with the closer the arrow is to the edge of the circle, the better the trait is represented. The functional traits are colored based on their measurement difficulty

From the PCA analysis on the six traits, we chose to keep the first 3 dimensions (or Principal Components), because all their eigenvalues were greater than 1, and summed up to a total of 76.6% of the original dataset inertia. According to the square cosine, the two hard traits were well represented by the first two PCA dimensions, capturing together 89% of the variation among strains in oxygen consumption and 77% in population growth rate (See Additional file 4: Table S2). For both traits, dimension 3 did not provide additional representation. On the first two dimensions, the other traits were represented at: 78% for NGDR, 41% for speed, 62% for cell size and only 5% for cell shape. Due to the poor representation of cell speed and shape, we could not assess their relations with the two hard traits. These dimensions showed a weak correlation between cell size and oxygen consumption, and a strong correlation between NGDR and population growth rate (Fig. 4A and B). Otherwise, the traits seemed rather independent from each other when plotted on the two first dimensions. To assess the relation between cell shape and speed, we plotted dimensions 1 and 3 together to maximize their representation (See Additional file 4: Table S2). On this plot, only three variables reached a square cosine higher than 50% and thus can be confidently assessed: NGDR (64%), speed (63%) and cell shape (85%). Among those, none displayed strong relations with each other (Fig. 4C and D). In conclusion, the PCA did not show any significant relations between three or more traits. Furthermore, even if the data were summarized with only three dimensions, within that space each trait was showing little redundancy with the others, meaning that including more than one trait in the models aimed at predicting other traits would not improve the fit.

## Discussion

Studies on functional diversity have encountered several challenges over the years [18, 51–53], such as measuring dozens of traits and choosing which traits to measure. In this study, we aimed to assess the relationship between several functional traits of a protozoan, *T. thermophila*, using a framework proposed by Hodgson et al. (1999) to deal with the challenge of measuring multiple traits, the soft/hard framework. Within this framework, one uses soft traits that are easy to measure (but not always the more meaningful in terms of ecological functionality) to predict hard traits that are functionally very desirable but difficult to obtain. The soft/hard framework assumes that the functional space can be reduced to a small number of traits, and so the presence of strong trade-offs or relations among traits. This is definitely an appealing framework that theoretically has the power to simplify the assessment of functional diversity, which might, in turn, be a key feature to understand the functioning of ecosystems. However, its utility clearly depends on how expected relations between traits are real and simple, because most of the statistical methods commonly used with this framework assume the relations between the traits to be monotonic and linear [54]. If this does not turn out to be true for many species, it might be the reason for the mixed results of applying this framework as of today in functional ecology studies [55, 56]. Here we tested this with a study system, *T. thermophila* in microcosms, for which knowledge is abundant enough to make informed predictions on correlations between a set of six functional traits, but also for which these predictions can be tested with good statistical power.

We detected several significant pairwise relations between the soft and hard traits, with over 60% of them being non-linear. Thus, the non-linearity in relations is a common phenomenon, which largely limited the ability to correctly conclude about the existence of relations between the traits using classical methods assuming simple linear relations, such as linear model and PCA. These results support the idea that, despite those methods being standard when it comes to detecting relations between variables, they should not be used without checking the linearity assumption first [54, 57, 58]. Still, even with a method that does not assume linearity (i.e. GAM), we could not find pairwise relations strong enough to allow reliable predictions of one trait using another. We suggest two possible causes for such absence of “good” relations -i.e. which meet the requirements detailed above: (i) a form that allows prediction and (ii) sufficiently low standard deviation of parameters, both resulting in a high deviance explained by the model.

The first one is an absence of the expected relations between these functional traits in *T. thermophila*. In theory, several phenomena can lead to a positive or negative relation between traits, like trade-off [59–61] (i.e. when one trait cannot increase without a decrease in another) or when traits are involved in the same function [62]. All of this limit the possibilities of trait association [63] and lead to a phenotype made of a whole suite of interrelated or coadapted traits, that one can define as the organism strategy [64]. At first sight, our data on *T. thermophila* fits this idea of strategy, with three dimensions representing more than 75% of the variability of six functional traits. However, a closer look at the PCA analysis does not show any clusters of more than two traits (Fig. 4A and C), with the link between those traits remaining weak, and the strains being scattered over the multivariate space (Fig. 4B and D). Even if the non-linearity sometimes improved the assessment of the link between traits, the GAM and the linear model both only displayed weak relations between traits, and again an absence of any cluster of more than two traits when we compared the models with each other. For example, the models using cell size, shape and speed as predictors explained between 20 and 40% of NGDR value; however, they were not good predictors of each other, despite all being linked to the same trait (i.e. NGDR). Thus, one could conclude that despite an important knowledge to express informed predictions about relations between traits, there is simply no straight and simple relations between the functional traits in our data. The fact that some of our informed predictions were already partly contradictory (Fig. 2) illustrates this complexity.

The second possible cause is the presence of factors that complexify the correct assessment of the relations between traits. In this paper, we focused on tackling the problem of the non-linearity between traits, since it is a basic assumption of statistical methods most often used to test for relations among variables [64–66]. We demonstrated that taking into consideration this non-linearity can significantly improve the prediction of one trait from another (Fig. 3). However, other factors can also blur the relations between traits in the analysis, with for example the plasticity of the traits [62, 64], their phenology [64, 67], the absence of a trait in the analysis [64], and the time scale considered [67]. Besides, these factors can also add up. Among those different examples, plasticity could have played a role in this study, since several traits of this species have been proven to be plastic [34, 46], even if we tried to control for it by standardizing the environment through the whole experimental process. In that case, the values of any functional trait could have varied among replicates, depending on (i) the trait considered, (ii) the magnitude of the differences in the experienced environment and (iii) the genetics of the strains. This variation of the trait values, induced by plasticity, might have loosened the relation between traits, or introduced a delay for the change of value in one trait to catch up with the other plastic traits it is tied to. In that case, it would not mean that there are no relations between traits within the species, but instead that they are blurred by some extra factors creating noise in our data.

Besides, this introduction of plasticity in the field of functional traits constitutes another reason for considering the non-linearity, since the relations between traits and environmental variables (i.e. reaction norms) are often non-linear (e.g. [34, 68]), and often follow a humped shape with an optimum, or a sigmoidal shape, reflecting the existence of environmental thresholds beyond which the trait performance changes drastically [69]. These non-linear relations might be rather common in nature [54], as between environmental variables and demographic rates (e.g. emperor penguin adult survival and sea ice concentration [70], Eurasian Oystercatcher fecundity and resource availability [71], red kangaroo survival and rainfall [72]). It is likely that the non-linear relations among traits are also very frequent, and assessing their prevalence across species is an avenue for future research. Importantly, should the commonness of non-linear relations among traits and environmental variables be confirmed across a broader range of taxa and locations, this would imply that the use of non-linear statistical approaches instead of basic correlations and PCA are needed for reliable quantification of functional diversity. The tools to analyze such non-linear relations become recently available, for example the newly developed senlm R package [73].

## Conclusions

Despite a set of informed predictions about expected relations between soft and hard life-history traits and a high statistical power resulting from a large number of *T. thermophila* strains and replicates, our study failed to reveal any relations between traits that would allow predicting one trait from another. However, this does not imply there is no relation between these traits in reality. It is possible that relations only appear or change in specific environmental conditions [55, 74] due to trait plasticity and/or environmental impact on trade-offs [15]; for an effect of environmental conditions on trait relations in the species, see [34]. For example, if two traits rely on the same resources, which would be scarce in harsh conditions, then investment choices have to be made by the organisms to invest in one trait at the expense of the other [63, 66], tightening the negative relation between traits. On the contrary, if there are enough resources available to invest in several traits (such as in our experiment with food ad libitum), it might loosen the relation between those traits, making the use of the soft/hard framework more difficult. One should not assume that the relationship between the traits will stay the same across the whole range of viable environmental conditions.

Our findings corroborate the idea that relying on soft traits that are simple and easy to measure as indirect proxies [75–77], may not always grasp correctly the hard traits which are directly linked to ecosystem functioning, if one does not know how soft and hard traits are related in first place. Considering the potential existence of non-linear relations may improve the ability to predict one trait by the other but also increases the complexity in calibrating the relation between the traits and precludes simple conclusions, such as “if the soft proxy increases, the hard trait should too”. Still, we believe that our study illustrates convincingly that one should not look for simplicity at all costs [68], but more for a reasoned simplicity, which is backed up by the investigations of what shortcuts could be taken to, for example, represent one functional trait by the other as a proxy.

## Methods

### Culture conditions and experimental design

All strains were maintained, before and during the experiment, under standardized culture conditions that allow only clonal reproduction [78, 79]. This involved axenic liquid culture in a nutrient medium (consisting of 2% Proteose peptone and 0.2% yeast extract, diluted in ultrapure water), kept at constant 27 °C temperature under a 14:10 h light/dark cycle. The stability of culture conditions is an important requirement for both the experiment and the maintenance of the cultures since *T. thermophila* shows a high degree of plasticity when the environmental conditions change [23, 33, 34]. Culture stocks were renewed/transferred every seven days by inoculating a 2 mL sample of fresh medium with 20 μL of the old culture and maintained in 24-multiwell plates (ref: 662102, Greiner BioOne). All manipulations of axenic cultures were conducted under sterile conditions in a laminar flow hood (Ultrasafe 218 S, Faster).

The experimental design involved measuring the six traits of interest for the 40 strains with a number of replicates that allowed reaching adequate statistical power for the analyses. The number of replicates was therefore a priori set to take into account the intrinsic error with which each trait can be measured in our microcosm setup, different for each trait, and slightly boosted to compensate for the limited but existing risk of losing some replicates due to bacterial contamination of the culture or potential other technical problems: 18 for growth measurements (measured over 5 days); 10 for measuring both the morphology, movement (snapshot measures) and oxygen consumption (measured over 2 h). For each strain, replicates each originated from different mother cultures, created from the stock culture three days before the experiment. This way of proceeding ensured that the replicates reflected the natural variability within each strain. To detect bacterial and fungal contamination, which can put cells under stress and alter their traits, we ran contamination tests after each trait measurement. These contamination tests were done by inoculating a Petri Dish containing a nutrient medium (Bacto Tryptone 2.5 g, Yeast extract 1.25 g, Glucose 0.5 g, Bacto Agar 9 g) with a few drops of the experimental cultures; contamination was detected by the presence of some fungal or bacterial development after three days at 27 °C. Contaminated samples were discarded from the analyses. We first created mother cultures for each strain and replicate by putting 500 µL of the corresponding culture stock and 6 mL of fresh axenic medium in a 30 mL cell culture container (ref: 201170, Greiner BioOne). Then, each mother culture was placed horizontally to favor oxygen ingress into the medium, and thus population growth. After a 3-day growth period, the measure of the six traits was performed on each replicate according to the protocols detailed below.

### Trait measurement

Morphology and movement traits were measured from digital images and videos recorded under a dark field microscope [47, 80] by placing two 10 µL samples from each mother culture into individual chambers on counting slides (ref: 630-2048, VWR). We took one picture for each chamber and one 20 s video of one of the two chambers, randomly chosen. For all identified cells from the two pictures, cell size (its area in µm^2^) and shape (i.e. aspect ratio: major/minor ratio of the two axes of a fitted ellipse; minimum is 1 for round cells, > 1 for more elongated cells) were quantified [80]. From the video, we estimated cell speed (in μm/s) and trajectory tortuosity (as net to gross displacement ratio, NGDR) using the BEMOVI R-package [81]. NGDR is computed as a ratio of the net displacement (i.e. straight line between the starting and the ending position) over the gross displacement (i.e. the total traveled distance during the measurement), meaning NGRD is 1 for a perfectly straight trajectory and < 1 for more tortuous ones.

Oxygen consumption was measured by quantifying the decay in dissolved oxygen concentration in a sealed high-density culture. Cell abundance was quantified in each mother culture (i.e. one replicate of a given strain) using a two-step process. In a first step, by measuring optical density at 550 nm (Genesys 20 spectrophotometer, Thermo Fisher Scientific), we were able to estimate the absorbance of the culture. In a second step, we combined that absorbance along with other factors (e.g. cell size) obtained from the pictures taken previously to estimate the cell abundance in the culture [47, 80]. This allowed us to standardize the experimental cultures at 1.5 mL and 200,000 cells/mL through dilution. Then, the experimental cultures were loaded into a 24-multiwell plate containing an individual oxygen sensor in each well (Oxodish OD24, PreSens). This plate was placed on a reader device (SDR SensorDish Reader, PreSens), and its 24 wells were sealed by covering the plate (without its lid) with a silicone mat and pressing the whole system (reader, plate and mat) between two plastic plates screwed together. As soon as the plate was sealed, the oxygen stopped flowing into the well, and we started recording the concentration of dissolved oxygen (in mg/L) within the experimental cultures every 2 min for 2 h. The measure of oxygen concentration using this technique is very sensitive to temperature, so the temperature was kept at precisely 27 °C during all steps, from the mother cultures to the whole measurement phase, and the temperature was recorded together with each measurement of oxygen concentration as a further check. After a short lag period, the recorded oxygen concentration started a linear decrease as the cells consumed oxygen, until reaching an asymptote below 20% oxygen saturation when almost all of the oxygen was consumed. As the trait quantifying oxygen consumption, we used the slope (in mg/L*min) of the linear decrease estimated using linear regression in the R software v3.6.1 [82].

To measure population growth rate, we created 2 mL experimental cultures by diluting each mother culture (i.e. one replicate of a given strain) in its stationary phase by a factor of five, to allow exponential growth; experimental cultures were placed in 24-multiwell plates (ref: 662102, Greiner BioOne). Cell density was estimated every two hours over five days using optical density at 550 nm (Synergy H1 microplate reader with robotic plate feeder, Biotek). The growth rate µ of each experimental culture was estimated as the slope of the optical density increase over time in its linear phase using the *gcfit* function (*grofit* R package [83]).

### Statistical analyses

To explore relations between the six traits, we averaged all replicates at the strain level because we were interested in functional traits exhibited by the strains, considered as the biological unit of replication, without integrating the within-strain variation between the replicates. This also compensated for the unequal number of replicates for the six traits. We were left with 51 discarded out of 721 replicates for growth and 18 discarded out of 400 replicates for morphology/movement/oxygen consumption, with a minimum replicate per strain of 12 for the growth and 8 for the other traits. All statistical analyses were performed on this set of 40 means at the strain level for each of the six traits. Morphology and movement traits, measured at the cell level, were in addition first averaged for every replicate to give the same weight to each replicate estimate irrespective of the number of cells identified on pictures and videos. Then, we performed two complementary analyses on our results.

In a first analysis, we explored the existence and linearity of pairwise relations between functional traits, by comparing a linear model (*glm* function, Gaussian distribution and identity link [82]) with a GAM model (*gam* function in *mgcv* R package, smoother estimation method: REML [84]). GAM are an extension of linear models that add nonparametric smooth functions to a model [85]. They are therefore used here as a test able to capture nonlinear patterns that a classic linear model would miss. Adding an extra bending point (or knot) in the GAM allows for a better fit (as adding an extra parameter in a linear model) but comes with a penalty, meaning that if the bending does not significantly improve the fit to the data (increasing the deviance explained), it will not be retained. The deviance explained by a model can be used to assess how well the model fits the data. To assess whether the non-linear relations fit the data better than the linear one we used the effective degree of freedom (e.d.f) provided by the GAM. Any e.d.f value above 1 indicates a significant non-linear relationship, and the higher the value, the more non-linear is the relation. We also explored whether fitting the linear model to prior transformed data would result in a better model fit. For this we have fitted linear models (Gaussian distribution and identity link) to log-transformed data, and compared the deviance explained by these models with the deviance explained by respective GAMs on non-transformed data.

In a second analysis, to examine the global dimensionality and test if the predictions of the models could be improved by combining several traits together, we conducted a principal component analysis (PCA) on standardized data (*FactoMineR* [86] and *factoextra* [87] R packages). To assess the individual representativeness of our traits on the dimensions, we used the squared cosine [57]. A high squared cosine indicates a good representation of the variable on the dimension under consideration. If a variable is perfectly represented by just two dimensions, the sum of squared cosine on these two axes is equal to 1, thus if we plot these two dimensions, the variable will be positioned directly on the correlation circle. Since our main goal was to study the relations between the hard and the soft traits, we used the square cosine to identify which dimensions to plot to best reflect the relations between our traits. However, unlike GAM, the main weakness of PCA here is that it needs simple linear relations between traits for the dimensionality reduction to be meaningful, hence the complementarity of the two approaches.

## Supplementary Information

**Additional file 1. Supplementary Figure 1.** Pairwise relationships among the six functional traits measured for the 39 *T.thermophila* strains, without the D14 which was suspected to be an outlier. Each dot represents the average valueof all replicates at the strain level, the blue line a linear regression, and the red curve a GAM. Both are representedwith their respective 95% confident interval. Above every graph is displayed the deviance explained (D.exp) byeach model. When outlined, the non-linear GAM is significantly better than the linear model (e.d.f. > 1), otherwisethe non-linearity does not improve the model’s fit and the GAM is behaving exactly as the linear model, hence thesame D.exp.

**Additional file 2. Supplementary Figure 2.** PCA analysis performed on the six functional traits (averaged at the strain level) for39 *T. thermophila* strains, without the D14 which was suspected to be an outlier. The left panel represent thedistribution of the strains along dimensions 1 and 2 (**A**) and along dimensions 1 and 3 (**C**). The numbers stand forthe labels of the strains. The right panel represent the associated correlation circles along the first and seconddimensions (*B*) and the first and third dimensions (**D**). The functional traits are colored based on their measurementdifficulty.

**Additional file 3. Supplementary Table 1.** Output of the Generalized Additive Models (G.A.M.) performed on relationshipsbetween the different traits of *T.thermophila*. The below diagonal part of the figure with white boxes correspondsto GAMs performed on the values of all 40 strains, while the above diagonal part with grey boxes corresponds toGAMs performed on only 39 strains, thus considering the strain D14 as an outlier.

**Additional file 4. Supplementary Table 2.** The square cosine of the different traits on the first three dimensions of the PCA. Thesquare cosine indicates how well a variable is represented on a considered dimension, and goes from 0 (the variableis not represented at all on that dimension) to 1 (the variable is completely represented on that dimension).

**Additional file 5. Supplementary Figure 3.** On the bottom left panel are displayed pairwise relationships among the six functionaltraits measured for the 40 *T. thermophila* strains. Each dot represents the log of the average value of all replicatesat the strain level, on which we fitted a linear regression, its predictions (together with 95% confidence interval)are shown in blue. Above every graph is displayed the deviance explained (D.exp) of the GLM on the logtransformeddata (in blue) and the GAM on the data before log transformation (in red) for comparison. The inseton the top right displays a boxplot of the deviance explained by those two methods, across all fitted pairwiserelationships, for comparison. On average, the GAM performed better.

## Data Availability

The datasets used and/or analyzed during the current study are available from the corresponding author on reasonable request.
