# Supplementary material for: Complexity vs linearity: relations between functional traits in a heterotrophic protist
Source: BMC Ecol Evol. 2023 Jan 11;23:1. doi: 10.1186/s12862-022-02102-w (PMC9832698; doi:10.1186/s12862-022-02102-w)
Supplement: Supplementary file 1 — Additional file 1. Supplementary Figure 1. Pairwise relationships among the six functional traits measured for the 39 T.thermophila strains, without the D14 which was suspected to be an outlier. Each dot represents the average valueof all replicates at the strain level, the blue line a linear regression, and the red curve a GAM. Both are representedwith their respective 95% confident interval. Above every graph is displayed the deviance explained (D.exp) byeach model. When outlined, the non-linear GAM is significantly better than the linear model (e.d.f. > 1), otherwisethe non-linearity does not improve the model’s fit and the GAM is behaving exactly as the linear model, hence thesame D.exp. [file 12862_2022_2102_MOESM1_ESM.pdf]

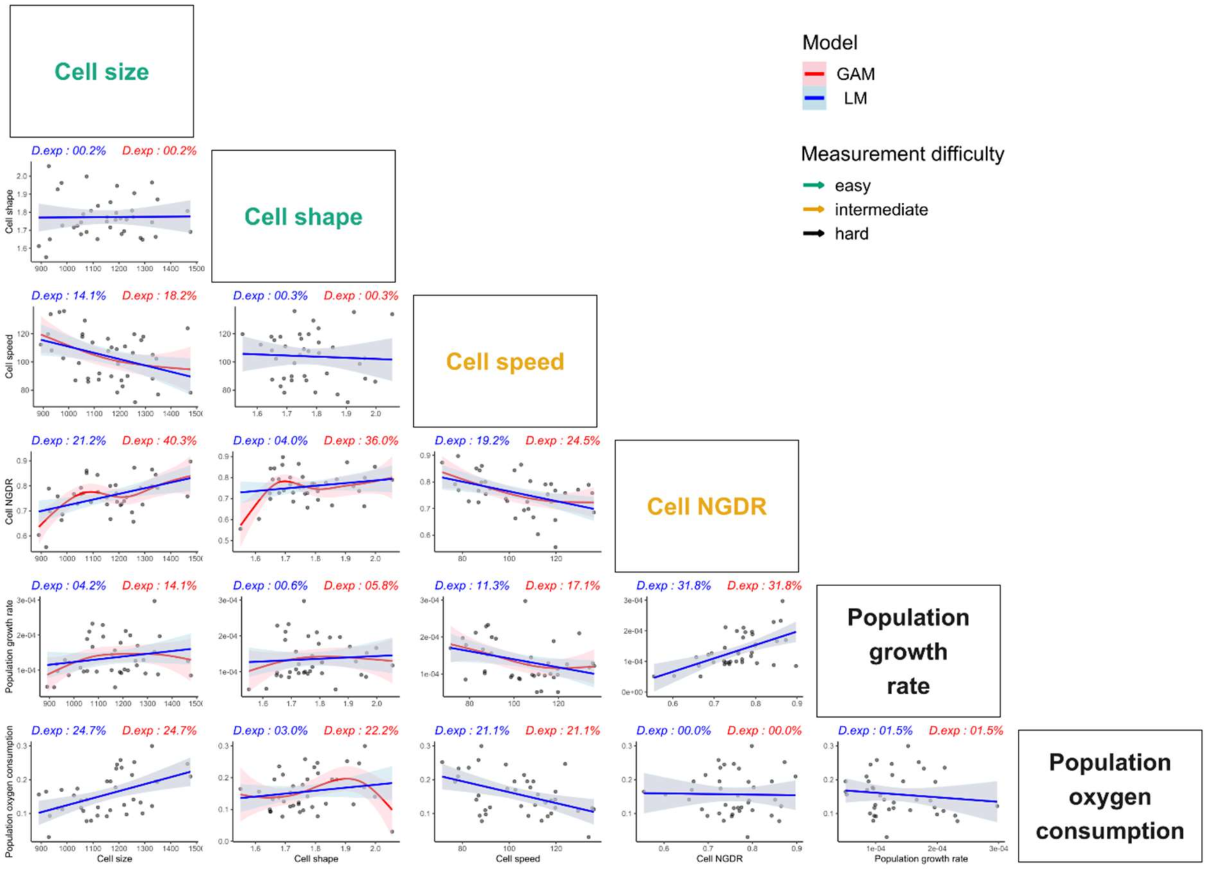

Supplementary Figure 1 – Pairwise relationships among the six functional traits measured for the 39 *T. thermophila* strains, without the D14 which was suspected to be an outlier. Each dot represents the average value of all replicates at the strain level, the blue line a linear regression, and the red curve a GAM. Both are represented with their respective 95% confident interval. Above every graph is displayed the deviance explained (D.exp) by each model. When outlined, the non-linear GAM is significantly better than the linear model (e.d.f. > 1), otherwise the non-linearity does not improve the model's fit and the GAM is behaving exactly as the linear model, hence the same D.exp.
