# Supplementary material for: Complexity vs linearity: relations between functional traits in a heterotrophic protist
Source: BMC Ecol Evol. 2023 Jan 11;23:1. doi: 10.1186/s12862-022-02102-w (PMC9832698; doi:10.1186/s12862-022-02102-w)
Supplement: Supplementary file 2 — Additional file 2. Supplementary Figure 2. PCA analysis performed on the six functional traits (averaged at the strain level) for39 T. thermophila strains, without the D14 which was suspected to be an outlier. The left panel represent thedistribution of the strains along dimensions 1 and 2 (A) and along dimensions 1 and 3 (C). The numbers stand forthe labels of the strains. The right panel represent the associated correlation circles along the first and seconddimensions (B) and the first and third dimensions (D). The functional traits are colored based on their measurementdifficulty. [file 12862_2022_2102_MOESM2_ESM.pdf]

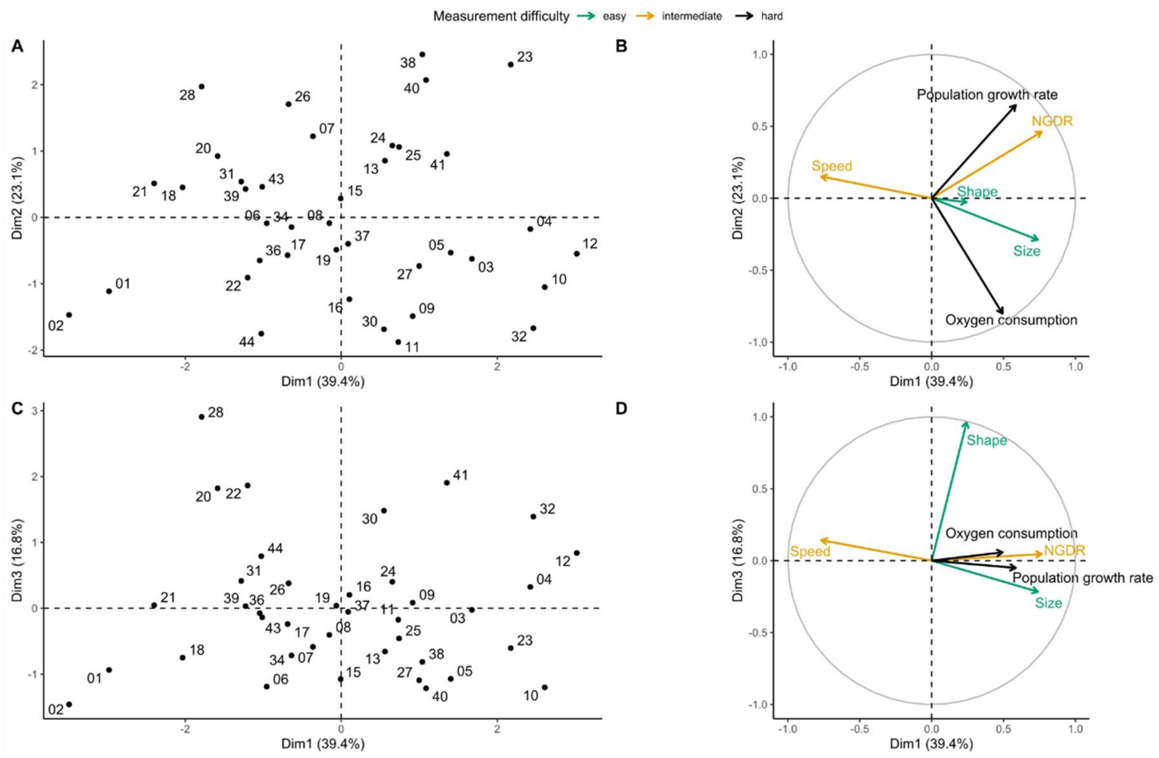

Supplementary Figure 2 – PCA analysis performed on the six functional traits (averaged at the strain level) for 39 *T. thermophila* strains, without the D14 which was suspected to be an outlier. The left panel represent the distribution of the strains along dimensions 1 and 2 (A) and along dimensions 1 and 3 (C). The numbers stand for the labels of the strains. The right panel represent the associated correlation circles along the first and second dimensions (B) and the first and third dimensions (D). The functional traits are colored based on their measurement difficulty.
