# Supplementary material for: Complexity vs linearity: relations between functional traits in a heterotrophic protist
Source: BMC Ecol Evol. 2023 Jan 11;23:1. doi: 10.1186/s12862-022-02102-w (PMC9832698; doi:10.1186/s12862-022-02102-w)
Supplement: Supplementary file 3 — Additional file 3. Supplementary Table 1. Output of the Generalized Additive Models (G.A.M.) performed on relationshipsbetween the different traits of T.thermophila. The below diagonal part of the figure with white boxes correspondsto GAMs performed on the values of all 40 strains, while the above diagonal part with grey boxes corresponds toGAMs performed on only 39 strains, thus considering the strain D14 as an outlier. [file 12862_2022_2102_MOESM3_ESM.pdf]

Y : Variable of interest	X : Explanatory variable					
	**Cell size**	0,86<br>1<br>0,02%	0,0354 *<br>1,64<br>18,20%	0,00342 **<br>3,61<br>40,30%	0,21<br>2,17<br>14,10%	0,00125 **<br>1<br>24,70%
	0,83<br>1<br>0,13%	**Cell shape**	0,75<br>1<br>0,28%	0,0237 *<br>4,23<br>36,00%	0,55<br>1,72<br>5,84%	0,15<br>3,3<br>22,20%
	0,25<br>1<br>3,47%	0.617<br>1<br>0,67%	**Cell speed**	0,0114 *<br>1,8<br>24,50%	0,07 •<br>1,85<br>17,10%	0,00325 **<br>1<br>21,10%
	0,0028 **<br>3,67<br>40,50%	0,0205 *<br>4,31<br>36,30%	0,0234 *<br>2,22<br>23,80%	**Cell NGDR**	0,000171 ***<br>1<br>31,80%	0,90<br>1<br>0,04%
	0,24<br>2,17<br>13,70%	0,58<br>1,76<br>5,70%	0,08 •<br>1,53<br>13,70%	0,000169 ***<br>1<br>31,20%	**Population growth rate**	0,45<br>1<br>1,54%
	0,00145 **<br>1<br>23,60%	0,24<br>2,98<br>17,80%	0,0283 *<br>1,82<br>20%	0,89<br>1<br>0,05%	0,47<br>1<br>1,39%	**Population oxygen consumption**

pvalue of the slope<br>number of e.d.f<br>% of deviance explained by the model

[white box] Values of the model with all the strains

[grey box] Values of the model without the possible outlier D14

Supplementary Table 1 – Output of the Generalized Additive Models (G.A.M.) performed on relationships between the different traits of *T.thermophila* . The below diagonal part of the figure with white boxes corresponds to GAMs performed on the values of all 40 strains, while the above diagonal part with grey boxes corresponds to GAMs performed on only 39 strains, thus considering the strain D14 as an outlier.

(•P<0.1, *P < 0.05, **P < 0.01, ***P < 0.001).
