# Supplementary material for: Complexity vs linearity: relations between functional traits in a heterotrophic protist
Source: BMC Ecol Evol. 2023 Jan 11;23:1. doi: 10.1186/s12862-022-02102-w (PMC9832698; doi:10.1186/s12862-022-02102-w)
Supplement: Supplementary file 4 — Additional file 4. Supplementary Table 2. The square cosine of the different traits on the first three dimensions of the PCA. Thesquare cosine indicates how well a variable is represented on a considered dimension, and goes from 0 (the variableis not represented at all on that dimension) to 1 (the variable is completely represented on that dimension). [file 12862_2022_2102_MOESM4_ESM.pdf]

	Dim.1	Dim.2	Dim.3
Size	**0.5**	**0.12**	**0**
Shape	**0.04**	**0**	**0.81**
Speed	**0.4**	**0.01**	**0.23**
NGDR	**0.61**	**0.17**	**0.03**
Population growth rate	**0.4**	**0.37**	**0.01**
Oxygen consumption	**0.2**	**0.69**	**0**

Supplementary Table 2 – The square cosine of the different traits on the first three dimensions of the PCA. The square cosine indicates how well a variable is represented on a considered dimension, and goes from 0 (the variable is not represented at all on that dimension) to 1 (the variable is completely represented on that dimension).
