# Supplementary material for: Complexity vs linearity: relations between functional traits in a heterotrophic protist
Source: BMC Ecol Evol. 2023 Jan 11;23:1. doi: 10.1186/s12862-022-02102-w (PMC9832698; doi:10.1186/s12862-022-02102-w)
Supplement: Supplementary file 5 — Additional file 5. Supplementary Figure 3. On the bottom left panel are displayed pairwise relationships among the six functionaltraits measured for the 40 T. thermophila strains. Each dot represents the log of the average value of all replicatesat the strain level, on which we fitted a linear regression, its predictions (together with 95% confidence interval)are shown in blue. Above every graph is displayed the deviance explained (D.exp) of the GLM on the logtransformeddata (in blue) and the GAM on the data before log transformation (in red) for comparison. The inseton the top right displays a boxplot of the deviance explained by those two methods, across all fitted pairwiserelationships, for comparison. On average, the GAM performed better. [file 12862_2022_2102_MOESM5_ESM.pdf]

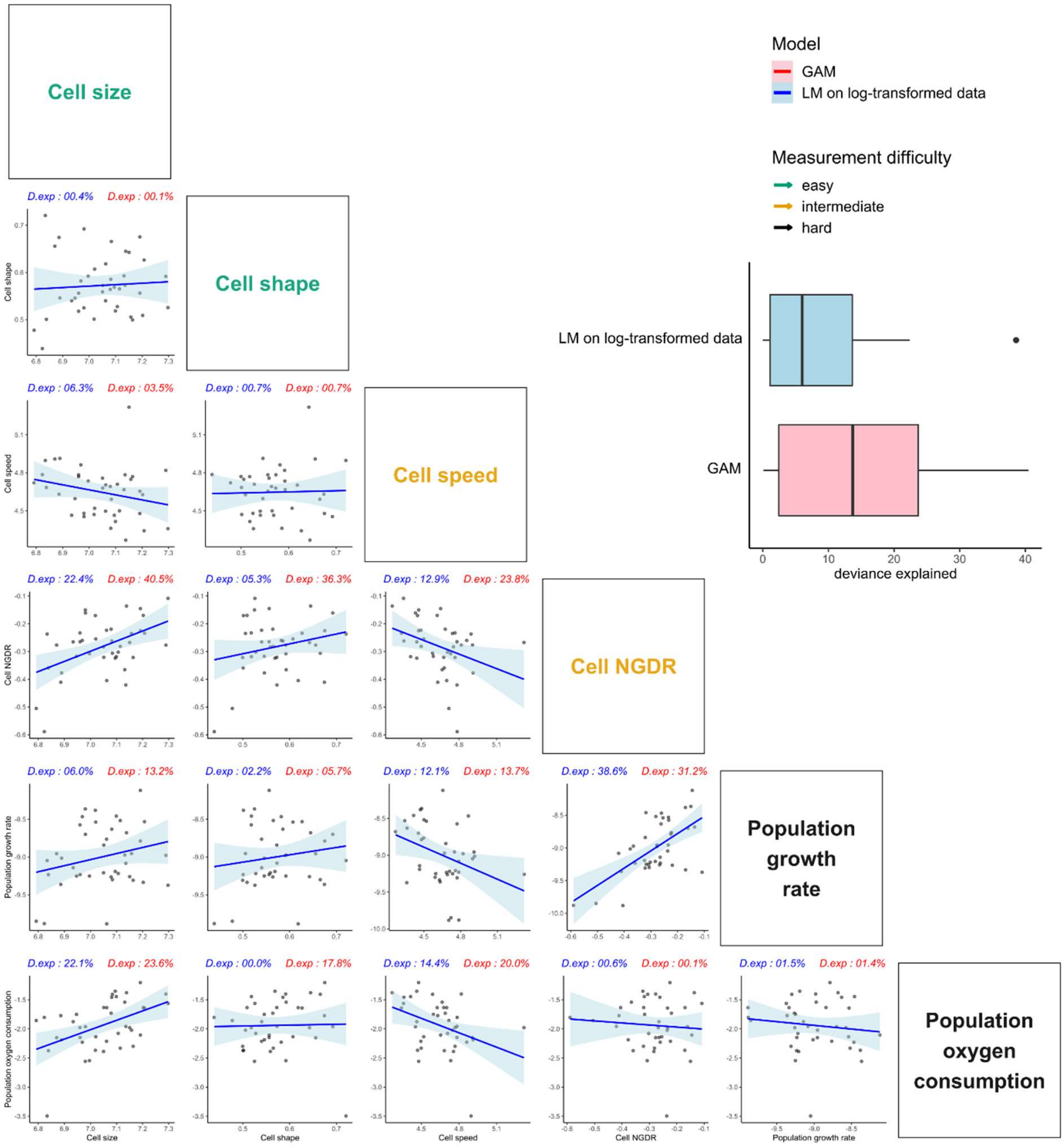

Supplementary Figure 3 – On the bottom left panel are displayed pairwise relationships among the six functional traits measured for the 40 *T. thermophila* strains. Each dot represents the log of the average value of all replicates at the strain level, on which we fitted a linear regression, its predictions (together with 95% confidence interval) are shown in blue. Above every graph is displayed the deviance explained (D.exp) of the GLM on the log-transformed data (in blue) and the GAM on the data before log transformation (in red) for comparison. The inset on the top right displays a boxplot of the deviance explained by those two methods, across all fitted pairwise relationships, for comparison. On average, the GAM performed better.
